# Greater glucagon-like peptide-1 responses to oral glucose are associated with lower central and peripheral blood pressures

**DOI:** 10.1186/s12933-019-0937-7

**Published:** 2019-10-05

**Authors:** Julie R. Lundgren, Kristine Færch, Daniel R. Witte, Anna E. Jonsson, Oluf Pedersen, Torben Hansen, Torsten Lauritzen, Jens J. Holst, Dorte Vistisen, Marit E. Jørgensen, Signe S. Torekov, Nanna B. Johansen

**Affiliations:** 1 Department of Biomedical Sciences, Faculty of Health and Medical Sciences, University of Copenhagen, Blegdamsvej 3B, 2200 Copenhagen N, Denmark; 2 Novo Nordisk Foundation Center for Basic Metabolic Research, University of Copenhagen, Copenhagen, Denmark; 3 Steno Diabetes Center Copenhagen, Gentofte, Denmark; 4 Aarhus University, Aarhus, Denmark; 5 Institute of Public Health, University of Aarhus, Aarhus, Denmark; 6 Danish Diabetes Academy, Odense, Denmark

**Keywords:** GLP-1, Cardiovascular disease, Central blood pressure, Peripheral blood pressure, Aortic stiffness

## Abstract

**Background and aim:**

Cardiovascular diseases (CVDs) are globally the leading cause of death and hypertension is a significant risk factor. Treatment with glucagon-like peptide-1 (GLP-1) receptor agonists has been associated with decreases in blood pressure and CVD risk. Our aim was to investigate the association between endogenous GLP-1 responses to oral glucose and peripheral and central haemodynamic measures in a population at risk of diabetes and CVD.

**Methods:**

This cross-sectional study included 837 Danish individuals from the ADDITION-PRO cohort (52% men, median (interquartile range) age 65.5 (59.8 to 70.7) years, BMI 26.1 (23.4 to 28.5) kg/m^2^, without antihypertensive treatment and known diabetes). All participants received an oral glucose tolerance test with measurements of GLP-1 at 0, 30 and 120 min. Aortic stiffness was assessed by pulse wave velocity (PWV). The associations between GLP-1 response and central and brachial blood pressure (BP) and PWV were assessed in linear regression models adjusting for age and sex.

**Results:**

A greater GLP-1 response was associated with lower central systolic and diastolic BP of − 1.17 mmHg (95% confidence interval (CI) − 2.07 to − 0.27 mmHg, P = 0.011) and − 0.74 mmHg (95% CI − 1.29 to − 0.18 mmHg, P = 0.009), respectively, as well as lower brachial systolic and diastolic BP of − 1.27 mmHg (95% CI − 2.20 to − 0.33 mmHg, P = 0.008) and − 1.00 (95% CI − 1.56 to − 0.44 mmHg, P = 0.001), respectively. PWV was not associated with GLP-1 release (P = 0.3). Individuals with the greatest quartile of GLP-1 response had clinically relevant lower BP measures compared to individuals with the lowest quartile of GLP-1 response (central systolic BP: − 4.94 (95% CI − 8.56 to − 1.31) mmHg, central diastolic BP: − 3.05 (95% CI − 5.29 to − 0.80) mmHg, brachial systolic BP: − 5.18 (95% CI − 8.94 to − 1.42) mmHg, and brachial diastolic BP: − 2.96 (95% CI − 5.26 to − 0.67) mmHg).

**Conclusion:**

Greater glucose-stimulated GLP-1 responses were associated with clinically relevant lower central and peripheral blood pressures, consistent with beneficial effects on the cardiovascular system and reduced risk of CVD and mortality.

*Trial registration* ClinicalTrials.gov Identifier: NCT00237549. Retrospectively registered 10 October 2005

## Background

Globally, cardiovascular diseases (CVDs) were the leading cause of death in 2017 [1]. Hypertension is a known risk factor of CVD and it is estimated that over 1 billion people suffers from hypertension [2]. Persons with uncontrolled hypertension are at increased risk of all-cause, CVD-specific, heart disease-specific and cerebrovascular disease deaths compared to normotensive persons (HR = 1.6, 2.2, 2.2 and 3.0, respectively) [3]. In recent clinical trials, treatment with glucagon-like peptide-1 receptor agonists (GLP-1 RAs) decreased cardiovascular morbidity and/or mortality in patients with type 2 diabetes at increased cardiovascular risk [4, 5]. Further, treatment with GLP-1 RAs decreases blood pressure and increases heart rate [6]. GLP-1 is an important incretin hormone which increases insulin secretion and thereby lowers blood glucose levels. It is secreted from the gastrointestinal tract by enteroendocrine L cells, upon nutritional intake, and is rapidly broken down by the enzyme dipeptidyl peptidase-4 (DPP-4) [7]. Beyond the insulinotropic effects, GLP-1 has numerous other effects including inhibition of glucagon secretion [8], inhibition of gastrointestinal secretion and motility [9] and reduction in appetite and food intake [10].

Chronic low-grade inflammation has been proposed to contribute to development of cardiovascular disease and atherosclerosis [11] and several inflammatory markers have been shown to be reduced by treatment with GLP-1 RA [12]. Based on a study with mice lacking the GLP-1 receptor (GLP-1R^−/−^) [13], it has been suggested that endogenous GLP-1 play a role in controlling the cardiovascular system [14]. This study showed that GLP-1R^−/−^ mice exhibited both structural and functional cardiac abnormalities compared to wild type mice. Another study in humans, found that acute GLP-1 infusion in physiological concentrations (corresponding to a postprandial level) did not change systolic blood pressure but did increase heart rate, brachial artery diameter and brachial artery flow velocity [15], indicating a physiologically relevant role of GLP-1 on vascular function.

Treatment studies with supra-physiological pharmacological doses of GLP-1 RAs have shown decreased risk of CVD [4, 5] and also a decrease in blood pressure [16] whereas the natural endogenous GLP-1 secretion and association with blood pressure measurements is largely unknown. Therefore, we wished to examine the more long term effects of endogenous GLP-1 response to elaborate on the cardiovascular mechanisms of GLP-1 and to investigate whether a greater endogenous GLP-1 response is associated with lower blood pressure. Hence, the aim of this study was to examine the association between endogenous GLP-1 response to glucose and peripheral and central hemodynamic measures in a large, Danish cohort of 837 individuals at risk of diabetes and thereby also at risk of cardiovascular disease.

## Methods

This cross-sectional study is part of a large observational cohort study: the ADDITION-PRO study, a longitudinal cohort study of individuals at high risk for diabetes, recruited from Danish primary care. A thorough description of the design can be found in the ADDITION-PRO protocol [17].

In the ADDITION-PRO study, a detailed examination programme was executed during 2009–2011, and 2082 individuals participated. Blood sampling for GLP-1 measurement was performed at three out of four study centres, comprising 1853 individuals. Of those, 252 had known type 2 diabetes and 15 did not fast and did not have an OGTT. Of the remaining 1586 participants, 657 were taking antihypertensive medication and 92 additional participants had missing information on central haemodynamics or GLP-1, leaving 837 individuals available for the present analysis. No individuals were using incretin-based drugs. Of the 837 individuals, 116 were using lipid-lowering drugs, which we allowed in the present analysis.

### Peripheral and central haemodynamics

Brachial blood pressure was measured three times with the participant in a sitting position after 10 min of rest (Omron M6 comfort, Omron Healthcare, Milton Keynes, UK) and reported as an average. Aortic pulse wave velocity (PWV) and central blood pressure were assessed with the participant in a supine position after 10 min of rest using a SphygmoCor apparatus (version 8, Atcor Medical, West Ryde, NSW, Australia) and a tonometer, which captured wave forms at the carotid, femoral and radial artery. Using simultaneous ECG monitoring, the time from the R wave to the arrival of the pulse wave at the carotid and femoral artery, respectively, was captured. An anthropometer (Seca, Medical Scales and Measuring Systems, Hamburg, Germany) was used to measure the distance from the suprasternal point to the carotid artery recording site and from the suprasternal point to the femoral artery recording site to avoid overestimation of the distance. The ratio between the distance and the time defined the PWV. The PWV was measured twice between the right carotid and right femoral artery and in case of a difference of more than 0.5 m/s, a third measure was taken. It was reported as an average of the two closest measures. Peripheral pressure waveforms assessed at the radial artery were used to estimate central blood pressure [17]. The average of two measures were used.

### Biochemical measures

A 3-point standard 75 g oral glucose tolerance test (OGTT) was performed, with blood sampling before (0 min) and 30 and 120 min after glucose intake. Plasma glucose was measured using one of two different methods; Hitachi 912 system (Roche Diagnostics, Mannheim, Germany) or Vitros 5600 system (Ortho Clinical Diagnostics, Illkirch Cedex, France). Subsequently, Vitros values were converted to Hitachi values [18]. HbA1c was measured by high-performance liquid chromatography (TOSOH G7, Tokyo, Japan) [18]. Plasma levels of total GLP-1 (intact and metabolites) were measured in EDTA plasma stored at − 80 °C using RIA with analytical detection limit around 1 pmol/l and with intra- and interassay coefficients of variation of 6.0% and 15%. To diminish methodological variances, all samples were analysed after study completion during 2 consecutive months. Furthermore, quality controls and batches for all reagents were identical for all sets [18].

### Statistics

GLP-1 response was defined as the incremental area under the curve (iAUC), from start of the OGTT to 120 min after glucose intake, using the GLP-1 measurements before glucose intake and after 30 and 120 min. Using the trapezoid rule, iAUC was calculated as total area under the curve subtracted the fasting GLP-1 value × 120 min (participants with iAUC ≤ 0 are excluded from analyses). Associations between GLP-1 release and central haemodynamics were analysed in separate models using linear regression analysis. Prior to analysis, iAUC for GLP-1(iAUC_GLP-1_) was log_2_-transformed to fulfil the requirement of a normal distribution of the model residuals. All analyses were adjusted for age and sex. Analyses of PWV were additionally adjusted for mean blood pressure and heart rate at time of PWV measurement. Additional analyses adjusting for age, sex and either iAUC insulin, fasting plasma glucose, BMI or HOMA-IR were performed for association between iAUC GLP-1 and central and brachial blood pressures. The study population was categorized into quartiles according to the iAUC GLP-1 response and the lowest and highest quartiles were compared with regards to blood pressure measures (adjusting for age and sex). Sub-analysis of the study population was made dividing the population into a normo glucose (fasting glucose < 5.6 mmol/l) group and a pre-diabetes risk (fasting glucose ≥ 5.6 mmol/l) group and association to blood pressures were investigated (adjusting for age and sex). Analyses were performed using SAS version 9.4 (SAS Institute, Cary, NC, USA) and figures were created in R version 3.2.2 (The R Foundation for Statistical Computing). A P-value of < 0.05 was considered significant.

## Results

**Fig. 1:**
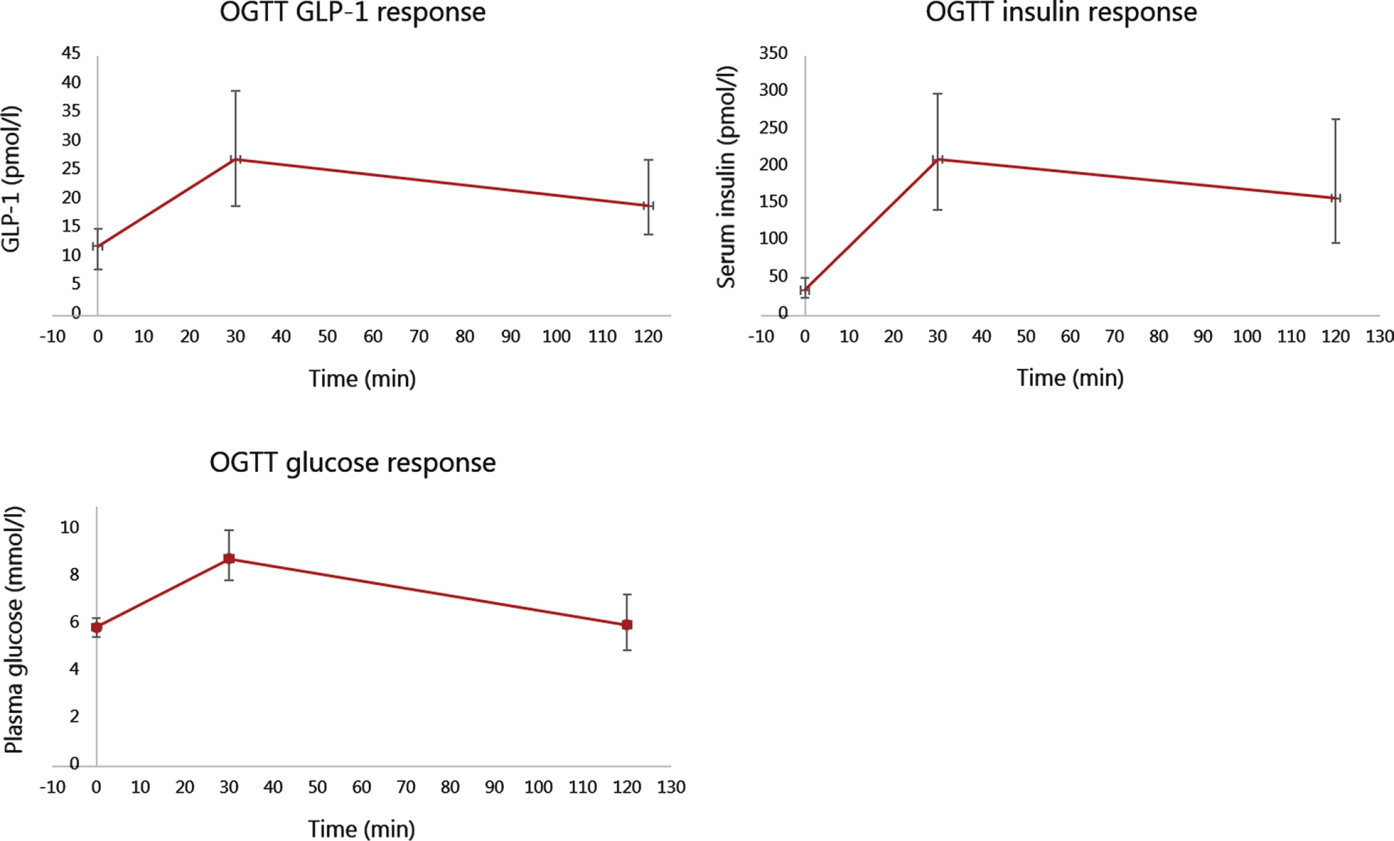
Oral glucose tolerance test (OGTT) responses of glucagon-like peptide-1 (GLP-1), glucose and insulin. Median is shown by a square and brackets show interquartile range

The 837 individuals included for analysis had a median (interquartile range) age of 65.5 (59.8 to 70.7) years and 47.7% were women, BMI of 26.1 (23.4 to 28.5) kg/m^2^, fasting glucose concentration of 5.9 (5.5 to 6.3) mmol/l, HbA1c level of 5.6 (5.4 to 5.8) % and a median blood pressure within the normal range (brachial systolic blood pressure of 130 (118.3 to 142) mmHg and diastolic blood pressure of 80 (73.3 to 87.0) mmHg), Table 1. OGTT responses of GLP-1, glucose and insulin are shown in Fig. 1.

**Table 1:** Characteristics

Characteristics	Median	IQR or n (%)
Age (years)	65.5	(59.8; 70.7)
Women, n (%)	399	(47.7)
BMI (kg/m^2^)	26.1	(23.4; 28.5)
Fasting plasma glucose (mmol/l)	5.9	(5.5; 6.3)
HbA1c (%)	5.6	(5.4; 5.8)
Fasting serum insulin (pmol/l)	34.0	(23.0; 51.0)
Fasting GLP-1 (pmol/l)	12.0	(8.0; 15.0)
Pulse wave velocity (m/s)	7.6	(6.6; 8.9)
Central systolic blood pressure (mmHg)	120.5	(110.3; 132.3)
Central diastolic blood pressure (mmHg)	78.0	(71.5; 85.0)
Central pulse pressure (mmHg)	41.5	(35.5; 50.2)
Brachial systolic blood pressure (mmHg)	130.3	(118.3; 142.0)
Brachial diastolic blood pressure (mmHg)	80.0	(73.3; 87.0)
Brachial pulse pressure (mmHg)	49.7	(41.7; 57.7)
Heart rate (beats/min)	61.3	(55.5; 67.8)

Characteristics of the study population. *IQR* interquartile range, *BMI* body mass index, *HbA1c* glycated haemoglobin, *GLP-1* glucagon-like peptide-1

**Fig. 2:**
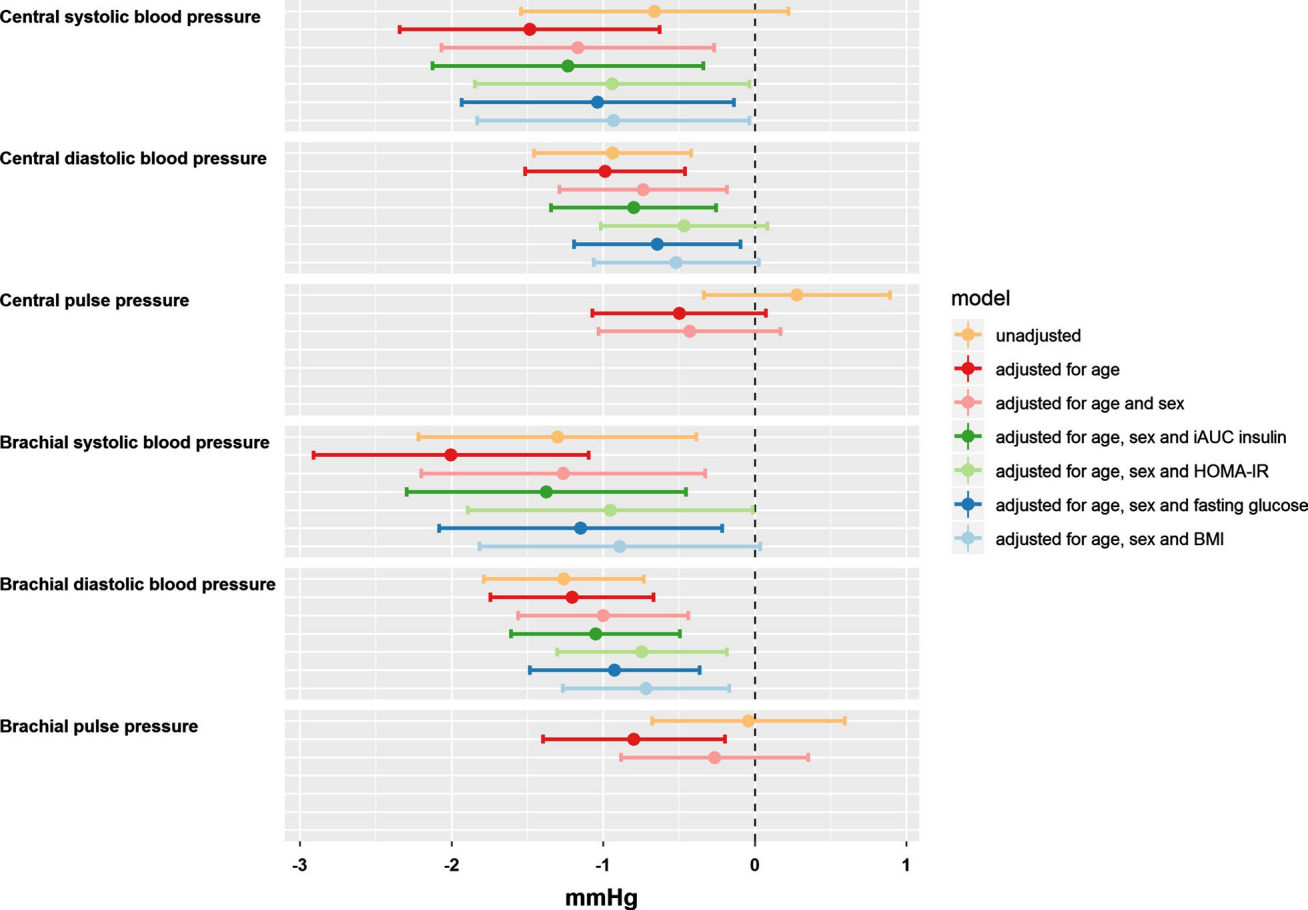
Estimated difference in blood pressure measures in 837 individuals for a doubling in incremental AUC (iAUC) of GLP-1. *HOMA-IR* homeostatic model assessment for insulin resistance, *BMI* body mass index

A greater GLP-1 response during the OGTT (iAUC_GLP-1_) was associated with a lower central systolic (− 1.17 mmHg; P = 0.011) and diastolic (− 0.74 mmHg; P = 0.009) blood pressure as well as a lower brachial systolic (− 1.27 mmHg; P = 0.008) and diastolic (− 1.00 mmHg; P = 0.001) blood pressure. Central pulse pressure and brachial pulse pressure were not significantly associated with GLP-1 secretion, Table 2 and Fig. 2.

**Table 2:** Haemodynamic measurements

Measurement	Estimate (mmHg)	95% confidence interval (mmHg)	P value
Central systolic blood pressure^a^	− 1.17	− 2.07 to − 0.27	*0.0109*
Central diastolic blood pressure^a^	− 0.74	− 1.29 to − 0.18	*0.0089*
Central pulse pressure^a^	− 0.43	− 1.03 to 0.17	0.2
Brachial systolic blood pressure^a^	− 1.27	− 2.20 to − 0.33	*0.0081*
Brachial diastolic blood pressure^a^	− 1.00	− 1.56 to − 0.44	*0.0005*
Brachial pulse pressure^a^	− 0.27	− 0.88 to 0.35	0.4
Pulse wave velocity^b^	− 0.04	− 0.13 to 0.04	0.3

Estimated difference in blood pressure measurements and PWV for a doubling in iAUC of GLP-1. P-values < 0.05 are italicized

^a^Adjusted for age and sex

^b^Additionally adjusted for heart rate and mean blood pressure during the PWV measurement

A doubling in iAUC_GLP-1_ resulted in a non-significant difference in PWV of − 0.04 m/s (P = 0.3).

Analyses were adjusted for age and sex and PWV analyses was additionally adjusted for blood pressure and heart rate. The association between GLP-1 response and central and peripheral blood pressure remained significant when adjusting for fasting glucose, iAUC insulin response, and for HOMA-IR (with the exception of central diastolic blood pressure) (in separate analyses). When adjusting for BMI, the association remained significant for central systolic blood pressure and peripheral diastolic blood pressure, Fig. 2. Sub-analysis of individuals with normal fasting glucose (fasting glucose level < 5.6 mmol/l, n = 270) and of individuals with pre-diabetes (fasting glucose level ≥ 5.6 mmol/l, n = 567) did not reach statistical significance and there was no significant difference between the groups.

### Greater GLP-1 response is associated with ~ 5 mmHg lower blood pressure compared to individuals with lower GLP-1 response

The participants were divided into quartiles according to their iAUC GLP-1 response. The group with the highest quartile of GLP-1 response was associated with significant lower central systolic (− 4.94 (95% CI − 8.56 to − 1.31) mmHg) and diastolic (− 3.05 (95% CI − 5.29 to − 0.80) mmHg) blood pressure as well as significant lower brachial systolic (− 5.18 (95% CI − 8.94 to − 1.42) mmHg) and diastolic (− 2.96 (95% CI − 5.26 to − 0.67) mmHg) blood pressure, compared to the group with the lowest quartile of GLP-1 response.

## Discussion

This study of 837 people at risk of diabetes and thereby CVD shows that there is a clinically relevant relationship between greater endogenous GLP-1 hormone response during an OGTT and lower blood pressure measures in a normotensive population. The quartile of individuals with the highest GLP-1 response had a ~ 5 mmHg lower blood pressure compared to the quartile of individuals with the lowest GLP-1 response. This degree of blood pressure lowering has been associated with lower risk of cardiovascular event, all-cause mortality and cardiovascular mortality. Thus indicating beneficial effects on the cardiovascular system with greater endogenous GLP-1 secretion in agreement with the observations of lower cardiovascular mortality and decreased blood pressure with GLP-1 RA treatment [4, 5, 16].

### Possible mechanisms of blood pressure lowering action of GLP-1 RA

Several parameters have been proposed to affect the secretion and concentration of GLP-1 including glucose [19], fat [19], bile acids [20], inflammatory markers [21, 22], 3-deoxyglucosone [23] (generated from carbohydrates) and the presence of obesity [24] and type 2 diabetes [18].

We find that there is an association between a greater GLP-1 response during an OGTT and lower central and peripheral blood pressure. Our results remain significant even after adjusting for iAUC insulin, fasting glucose, and for most analyses also for BMI and HOMA-IR, which are all factors known to have an impact on GLP-1 secretion. Interestingly, when dividing participants into quartiles according to their GLP-1 response we found that the group with the highest GLP-1 response was associated with approximately 5 mmHg lower blood pressures compared to the group with the lowest GLP-1 response. Many individuals with obesity and/or type 2 diabetes have decreased GLP-1 secretion [18, 24, 25] and these individuals also often have increased blood pressure [26, 27]. Furthermore, weight loss increases GLP-1 response and decreases blood pressure [25, 28]. Thus, according to our results, it could be speculated whether the decreased GLP-1 response could contribute to the increased blood pressure in this population. Together with our analyses adjusted for several parameters, this implies that endogenous GLP-1 may have an effect on blood pressure in a dose–response dependent manner with the greater the GLP-1 response the lower the blood pressure as also observed for the dose-response dependently lowering of blood glucose [29, 30].

### Clinical implications

We observe that the quartile of individuals with the highest GLP-1 response have a ~ 5 mmHg lower blood pressure compared to the quartile of individuals with the lowest GLP-1 response. A large meta-analysis found that the degree of blood pressure reduction was proportional to the risk of major cardiovascular disease events, stroke, heart failure, and all-cause mortality [31]. Interestingly, a reduction of 4.5 mmHg in systolic blood pressure has been associated with lower risk of cardiovascular event, all-cause mortality and cardiovascular mortality [32]. Thus our finding of a 5 mmHg difference in blood pressure between GLP-1 response groups is of clinical relevance with regards to mortality. In comparison, DPP-4 inhibitors reduce systolic blood pressure by 2.5–2.8 mmHg [33–35] and GLP-1 RAs reduce systolic blood pressure by 1.2–4 mmHg [4, 5, 36]. GLP-1 RAs [4, 5, 37] have, furthermore been found to reduce risk of major adverse cardiac events which has not been observed for DPP-4 inhibitors [38–40].

The GLP-1 receptor has been located on endothelial cells in the vasculature [41] and a direct effect of GLP-1 on vascular smooth muscle has also been proposed [42]. This may be one of the mechanisms by which GLP-1 RA treatment decreases blood pressure. Endothelial dysfunction, characterized by reduced nitric oxide (NO) production, is a precursor of atherosclerosis and a risk factor for CVD [43]. GLP-1 treatment promotes vasodilation by production of NO [44]. Insulin has also been shown to induce NO production [45], however, the association in our study remained significant when adjusting the analyses for insulin (see Fig. 2), indicating that GLP-1 works independently of insulin on the blood pressure-lowering mechanism. Infusion of GLP-1 in physiological doses exerted vasodilatory actions, decreased diastolic blood pressure and increased heart rate whereas systolic blood pressure was unchanged in a study of 26 healthy individuals [15]. Furthermore, treatment with GLP-1 RAs have shown to improve arterial stiffness in type 2 diabetes [46], which is augmented by hypertension [47]. Similarly, treatment with DPP-4 inhibitors have shown to improve intima-media thickness [48], arterial stiffness [49] and diastolic dysfunction [50, 51], which are all factors associated with blood pressure [52, 53]. In our study of individuals without diabetes and hypertension we did not find an association between GLP-1 response and PWV (Table 2), a measurement of arterial stiffness, or central pulse pressure (Fig. 2), which is determined by arterial stiffness [47]. To further support the effect of GLP-1 RAs on endothelial cells, liraglutide has also been found to have anti-restenotic effects mediated by endothelial NO [54]. Endothelial cells do not only play a role in NO production, they also influence on the angiogenesis process [55] and liraglutide has been found to promote angiogenesis after stroke [56] opening up for the possibility that GLP-1-based treatment potentially could be applied as a neuroprotective drug as well as a cardioprotective, anti-hyperglycemic and anti-obesity drug.

### Other studies with endogenous GLP-1 levels

Only few studies have previously analysed the association between endogenous GLP-1 levels and cardiovascular disease. In agreement with our findings, an inverse correlation (r = − 0.26, P = 0.0085) between GLP-1 secretion and systolic blood pressure was found by Yoshihara et al. [57]. However, active GLP-1 (the levels of which are most often below detection levels of the assays) rather than total GLP-1 was measured in this study of 103 individuals with an ELISA kit. Another study reported a positive association between levels of GLP-1 and coronary artery disease [58]. However blood samples were gathered randomly, i.e. not necessarily in a fasting state, from 303 persons and GLP-1 levels (active GLP-1 and metabolites) were measured by ELISA in serum. Since random blood sampling is a major limitation to the study above comparability to other studies is likely to be difficult. Furthermore, it is worth noting that the specificity, sensitivity, and validity can vary considerably between the ELISA kits available for GLP-1 analyses [59].

### Strengths and limitations

Our physiological study includes more than 800 participants making its size a major strength, outnumbering previous studies [57, 58]. Total GLP-1 (not active/intact GLP-1) was measured with the use of a well-documented RIA with high specificity and sensitivity [60]. Measuring GLP-1 in this manner provides more information than solely measuring intact GLP-1 as GLP-1 metabolites also are included in the analyses [18]. Because of the rapid metabolism of the hormone, estimation of its secretion must be made with an assay for total GLP-1. A limitation of this study, however, is the cross-sectional design, allowing us to find associations between GLP-1 and central and peripheral haemodynamic measures whereas we cannot conclude on causality. However, our findings are in agreement with observations from GLP-1 treatment studies on lowering hypertension and cardiovascular mortality [4, 5]. Haemodynamic measurements were collected only in a fasting state. Therefore, it was not possible to investigate the acute effects of GLP-1 secretion on blood pressure and PWV. Other studies have found no change [61] or increase [62] in blood pressure after acute infusion of GLP-1. In general, there does not seem to be consensus about whether blood pressure changes during an OGTT. One study found an elevation in systolic blood pressure of more than 4 mmHg after glucose load [63] while other studies find either small increases [64] or no differences [65]. Lastly, our sub-analysis dividing participants into a normal fasting glucose group and a pre-diabetes group did not reveal any significant differences between the groups, which may be due to the lower number of participants in each group.

## Conclusion

We found that greater glucose-stimulated GLP-1 responses were associated with clinically relevant lower central and peripheral blood pressure. Thus, it is likely that endogenous GLP-1 response has a beneficial impact on vascular function, consistent with beneficial effects on the cardiovascular system and reduced risk of CVD and mortality.

## Data Availability

The dataset used and analysed during the current study are available from the corresponding author on request.

## Abbreviations

BDBP: brachial diastolic blood pressure
BMI: body mass index
BP: blood pressure
BPP: brachial pulse pressure
BSBP: brachial systolic blood pressure
CDBP: central diastolic blood pressure
CI: confidence interval
CPP: central pulse pressure
CSBP: central systolic blood pressure
CVD: cardiovascular disease
DPP-4: dipeptidyl peptidase-4
GLP-1: glucagon-like peptide-1
GLP-1 RA: glucagon-like peptide-1 receptor agonist
HOMA-IR: homeostatic model assessment for insulin resistance
HR: heart rate
iAUC: incremental area under the curve
NO: nitric oxide
OGTT: oral glucose tolerance test
PWV: pulse wave velocity
